# Atrial natriuretic peptide (ANP) and oxytocin-expression in the adult rat and mouse cerebellum

**DOI:** 10.1186/s40673-015-0031-1

**Published:** 2015-10-06

**Authors:** Alessio Lipari, Elvira Farina, Aldo Gerbino, Luana Lipari

**Affiliations:** Department of Experimental Biomedicine and Clinical Neurosciences, University of Palermo, Via del Vespro, 129, 90127 Palermo, Italy

**Keywords:** Cerebellum, ANP, Oxytocin, Immunohistochemistry, Rat, Mouse

## Abstract

**Background:**

Many studies are in the literature on the ANP and oxytocin-presence in the brain, but very few studies with controversial results are reported on the presence of these peptides in the cerebellum. This immunohistochemical study investigates on the ANP and oxytocin-presence in the cerebellum of the adult rat and mouse rodents.

**Results:**

This study, firstly, evidences the ANP- immunopositivity in cerebellar cortex of both rat and mouse rodents. In rat the molecular layer presents some few immunopositive fibers, but no neuron resulted immunopositive; the granular and Purkinje cells are immunopositive. In mouse the cerebellar cortex ANP-immunopositivity is present in all layers. The oxytocin-presence in the rat the afferent fibers are immunopositive are in the granular layer; in mouse the OT-immunopositivity is in the molecular layer only.

**Conclusions:**

This study, firstly, shows that ANP and OT are present in the cerebellar cortex both in rat and mouse rodents. In the mouse cerebellar cortex ANP-presence is more diffuse and OT- localization differences in the two species.

## Background

Atrial natriuretic peptide (ANP) is a peptide synthesized in the heart, but it is known that ANP is present in different peripheral organs. In rat brain ANP was showed be in the septum and hypothalamus (1) and also ANP-binding sites were evidenced in different parts of brain. The presence of ANP and ANP-binding sites the mammalian brain suggests that the ANP could act as a modulator of non cardiovascular functions.

In the mammalian cerebellum it was evidenced that both ANP peptide and its specific receptors appear to species specific heterogeneity. In rat cerebellum no ANP-positive cell was found [1]; regarding the ANP-binding sites the results are controversial, indeed Gibson et al. [2], by autoradiography, reported the presence of the ANP-binding sites in low density, while Manthy [3] reported the absence of ANP-binding sites.

In guinea pig ANP-binding sites are present in granule cell layer of cerebellum [3] and also in monkey cerebellum [4].

In the canine cerebellum ANP moderate levels, by radioimmunoassay, were found [5].

In the human cerebellum the ANP-like immunoreactivity was in climbing fibers and in protoplasmic and fibrous astrocytes and Bergmann glia, as well as Golgi and Lugaro neurons of the granule cell layer [6]. Since the studies reported controversial results regarding the ANP presence in the cerebellum of the different species, this our study aims to investigate, by immunohistochemical method, the presence and localization of ANP in adult rat and mouse cerebellum, since our opinion is that ANP may be involved in the modulation of some cerebellum activities, i.e.by paracrine/autocrine mechanisms.

In regard to oxytocin (OT) in the cerebellum no paper is reported.

## Methods

Adult animals were used in this study in accordance with the Guide for the Care and Use of Laboratory Animals and in accordance to a protocol approved by our institutions animal care ethics committee. Ten adult Wistar rats and ten adult mice were housed in cages, alternating 12 light/dark periods in temperature and humidity controlled room, with free access to food/water. From every anesthetized animal the cerebellum was removed, fixed in Bouin’s fluid, paraffin embedded and sections (7 μm) were cut. The slides were dewaxed in xylene and rehydrated in a graded series of alcohols and were then transferred into distilled water for 5 min. The immunostaining was performed using the “Dako Cytomation EN Vision + System-HPR (AEC)”. The sections were incubated with “peroxidase block” reagent for 5 min RT, were rinsed, once, in PBS buffer pH 7.2 and incubated with the Rabbit Anti-ANP polyclonal antibody (Chemicon, Temecula, CA, USA), at 1:800 dilution (0.1 %BSA) 4 °C or with Oxytocin-antibody (Chemicon, Temecula, CA, USA) at 1:600 dilution) in 0.05 M Tris buffer pH 7.2 overnight at 4 °C. Samples were rinsed, twice, in PBS pH 7.2, then incubated with the “Substrate- Chromogen” reagent and immediately observed under a light microscope; the reaction was stopped by rinsing in distilled water. Slides were coverslipped using the “Dako Cytomation Faramount Aqueous Mounting Medium”. Negative control samples were treated in the same way, however, by omission of the primary antibody.

## Results

**Fig. 1:**
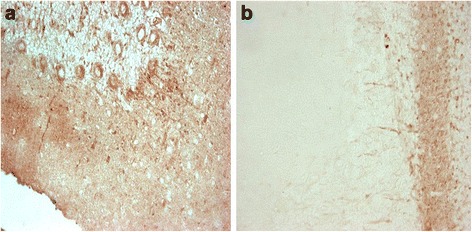
**a** Rat cerebellum: The Purkinje cells and their dendrites are ANP-immunopositive. Some immunopositive parallel fibers are visible. 40×. **b** Rat cerebellum: OT-immunopositive afferents fibers in the granular layer; the Purkinje cells are OT-immunonegative. 40×

ANP**-**immunostained sections showed that the rat cerebellum presents, into the lamellae of the white substance, numerous compacted fascicles of fibers that were strongly ANP-immunopositive; these fibers penetrated and subdivided in the granular layer, many fibers crossed the granular layer and their extremities surrounded the cell body of the Purkinje cells. In the rat cerebellar cortex the granular layer shows an intense positivity, the Purkinje cells are immunonegative in some areas, in other areas the Purkinje cells show ANP-immonopositivity in their cell body and dendrites projecting into the molecular layer; the molecular layer presented some few immunopositive fibers, but no neuron resulted ANP-immunopositive (Fig. 1a).

In rat cerebellum the afferents fibers are OT-immunopositive, the Purkinje cells are OT-immunonegative and, equally, all neurons and fibers in the molecular layer are OT-immunonegative (Fig. 1b).

**Fig. 2:**
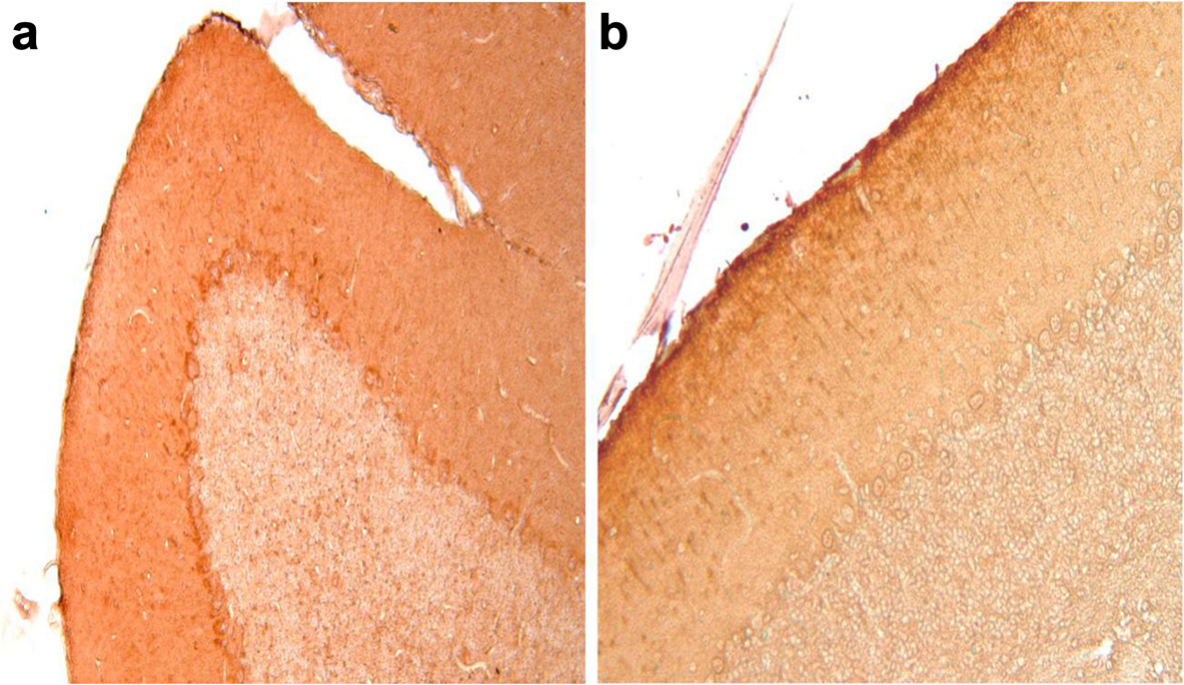
**a** Mouse cerebellum: In the granular, Purkinje cells and molecular layers ANP-immunopositive fibers and neurons are visible. 20×. **b** Mouse cerebellum: In the molecular layer the OT-immunopositive fibers and some neurons are immunopositive. The Purkinje cells and the granular layer are OT-immunonegative. 20×

In mouse the cerebellar cortex ANP-immunopositivity appears be more diffuse. Indeed, fibers in the lamellae of white substance are positive and in the cerebellar cortex the granular layer is immunopositive, the Purinkje cells are ANP-immunopositive in the perinuclear cytoplasm and, also, many fibers are present in the molecular layer (Fig. 2a).

In mouse cerebellum the OT-immunopositivity is only in the molecular layer where the immunopositive fibers oriented perpendicularly to the surface are OT-immunopositive are visible, and also some neurons present perinuclear OT-immunopositivity. The Purkinje cells and the granular layer are OT-immunonegative (Fig. 2b).

## Discussion and conclusions

In the rat brain the ANP-peptide was shown be distributed through whole brain with a very high concentration is in the hypothalamus and septum [1, 7]. CNP is also distributed widely in brain, but its level does not differ in each brain region [8]. In our previous studies showed in the developing rat that the ANP is present in the hypothalamic supraoptic [9], suprachiasmatic nuclei [10] and lateral choroid plexus [11], therefore also showed that ANP is involved in the resistance training, since in the hypothalamic supraoptic nucleus ANP-immunopositivity is lesser in the trained than the sedentary rats and the immunopositivity increases from 15th to 45th days of a resistance training with decreased release in bloodstream of the peptides, [12]; by contrast in the hypothalamic paraventricular nucleus the ANP-immunopositivity has no significant change [13].

In the mouse no study is reported, only Yeung et al. [14] found that the primary cultures of mouse astrocytes from cerebellum, hypothalamus and cerebral cortex can to bind the human ANP and porcine BPN by a single population of binding sites and that ANP and BNP also evoked cGMP stimulation in a similar, dose-dependent fashion in astrocytes from all three regions.

This study shows that ANP is present in the cerebellum both in rat and mouse rodents.

In the rat cerebellum the results show ANP-presence in disagreement with the results reported by Kawata et al. [1]. Furthermore regarding the ANP-location the results show that in the mouse cerebellum ANP-presence is more diffuse than in rat cerebellum. Indeed, fibers in the lamellae of white substance are positive and in the cerebellar cortex the granular layer is positive, the Purkinje cells are ANP-immunopositive in their perinuclear cytoplasm and, also, many ANP-immunopositive fibers are present in the molecular layer.

This indicates that the ANP not is confined to some neurons but ANP is involved into the intracerebellar circuits probably as a modulator transmitter. In conclusion, firstly this study shows that ANP is present in the cerebellum both in rat and mouse rodents.

In regard to OT, the studies are on the hypothalamic nuclei, but very few are studies on the OT-localization in the cerebellum, so our study shows the OT-presence in the rat and mouse cerebellum and evidenced that the OT-localization differences in the two species.

Furthermore since some recent studies demonstrated that the cerebellum regulates non only the motor coordination but also plays a cognitive function [15]; thus, it is possible think that OT plays an important role in the motor memory.
